# Piketty, Thunberg, or Marx? Shifting ideologies in the COVID-19 bailout conditionality debate

**DOI:** 10.1057/s42214-020-00072-8

**Published:** 2020-10-09

**Authors:** Elisa Giuliani

**Affiliations:** Responsible Management Research Center – REMARC, Department of Economics and Management, University of Pisa, Via Ridolfi 10, 56124 Pisa, Italy

**Keywords:** COVID-19, crisis, MNEs, bailout, sustainability, capitalism

## Abstract

Are crises an opportunity to push for fundamental changes? Can the COVID-19 crisis help to promote an ideological shift towards a different type of capitalism? By conducting a quantitative content analysis of the international press on the COVID-19 bailout conditionality debate, this article documents the existence of three dominant narratives: the distributive justice, environmental justice, and Marxist-type anti-capitalistic narratives. Yet, only the distributive justice assumed greater prominence during the period of observation, signaling a small step towards an ideological shift in which the general public may become more open to scrutiny of companies’ dividend and bonus policies and more averse to acceptance of tax avoidance and the accumulation of excessive wealth by members of the corporate elite. The article concludes by recommending MNEs and their CEOs to be prepared for more distributive justice challenges in the future.

Much has changed since the bubonic plague that hit England in the mid-1600s but, as the above quote argues, there is a common thread linking this earlier pandemic and the current COVID-19 crisis: both hit the poor much harder than the rich. Pandemics are only one of several sustainability grand challenges in need of a solution, whose impacts are especially severe for the underprivileged and poorer strata of our society. Other current challenges include a range of environmental threats from climate change to the noxious impacts of toxic pesticides; a wide spectrum of business-related human rights risks including those related to child labor and modern slavery; the rise of within-country economic inequality and the problem of tax avoidance.

While diverse in nature, all of these issues are global problems that need global solutions, and are challenges about which scientific research has produced significant knowledge. However, governments’ responses have often been slow, fragmented, and therefore ineffective on a global scale. By most accounts, these challenges represent ‘wicked problems’ (Rittel & Weber, 1973), which are hard to tackle because they are social systems problems (Churchman, 1967) around which decision-makers have conflicting values and whose complexity requires multi-stakeholder coordination and the involvement of public as well as private actors on a global scale. As suggested by Eden and Wagstaff (2020) these are problems where “politics trumps evidence and solutions are never first best or permanent”. Even in the most successful cases of implementation of global treaties to regulate and fix problems (e.g., the UN Declaration on Universal Human Rights, the Paris Agreement on Climate Change, and the Stockholm Convention on pesticides), not all countries have chosen to ratify these treaties at the same time or to enforce national regulation to ensure their success.

In international business terms, this fragmentation leads to institutional voids which allow companies with an international reach such as multinational enterprises (MNEs) to engage in social or environmental arbitrage practices in order to profit from imperfect global regulatory architectures (Surroca, Tribo & Zahra, 2013). It is interesting that rather than being condemned, these arbitrage practices are accepted as part of a portfolio of legitimate activities that MNEs can undertake to remain profitable, and which are openly exploited by governments to attract investors. In this context, international business ethics scholars have warned about the tensions that international managers will have to face when they operate in countries whose cultural or legal standards are different from those of their home country, especially if host countries’ legal requirements are loose or poorly enforced (Donaldson & Dunfee, 1999). Such studies have led to recommendations for managers to find ways to maintain high standards when it comes to fundamental rights, on issues like for instance “child labor, prison labor, or discrimination” (p. 60), (s.c. hypernorms), while otherwise they have warned about the need to find creative ways to adapt to and respect local cultural norms and habits. However, as widely documented by the business and human rights literature (see Giuliani & Macchi, 2014 for a review), it has proven very hard for MNEs to respect fundamental rights in poorly regulated host countries, where exploitative or otherwise-defined lower standard conduct may not even be considered unlawful or immoral. More fundamentally, however, arbitrage is tolerated because it is a practice that pays off economically: it greases MNEs’ operations and thus allows for local economic gains in the form of more and better jobs, and economic growth.

The point I want to make is that the narrative that endorses MNEs’ regulatory arbitrage in the name of economic value generation is not based on inviolable economic principles. Rather, I would argue that its acceptance is based on the decision-makers’ cognitive frames and reflected in the dominant narratives that have been constructed around given issues which condition ideologies and thus lead to the acceptance of certain “unitary truths” (Smith & Tushman, 2005) and to the rejection of equally valid or even potentially superior other “truths”. Research in fields as diverse as e.g., linguistics (Fillmore, 1976; Lakoff, 2004) and strategic change (Fiss & Zajac, 2006) shows that cognitive frames – the way in which individuals make sense of the complex world around them – can be performative, meaning that they can shape actions and decisions to change (Cornelissen & Werner, 2014). In economics, Douglass North has provided a fascinating account of the relevance of beliefs and ideologies for institutional development and economic growth. As he put it: “the dominant beliefs, that is, of those political and economic entrepreneurs in a position to make policies, over time result in the accretion of an elaborate structure of institutions, both formal rules and informal norms, that together determine economic and political performance.” (North, 2003, p. 4).

However, accomplishing such changes requires a cognitive frame shift, and this does not come easy. For example, decision-making around sustainability issues is an area where resistance to change (Ford, Ford, & D’Amelio, 2008) is particularly severe because it involves disruption to comfort zones and acceptance of paradoxes and ambiguities to which most decision-makers are reluctant to agree (Hahn, Preuss, Pinkse, & Figge, 2014). On this front, it seems that the current dominant cognitive frame is still one where sustainability goals are a desirable outcome only if their achievement is not detrimental to economic performance, and for this reason win–win business models – where sustainability projects are used instrumentally to increase profits – have become a very popular way of addressing sustainability challenges (Van der Byl & Slawinski, 2015). However, win–win models often imply the perseverance of the standard profit-oriented cognitive frames, and neither embrace new more paradoxical logics nor imply radical departures from ‘business as usual’ (Giuliani, Tuan, & Calvimontes Cano, 2020).

## THE NARRATIVES IN THE BAILOUT CONDITIONALITY DEBATE DURING THE COVID-19 CRISIS

If we look at the past, crises potentially can spark some changes to the dominant narratives and to policymaking. Whereas on the one hand it is true that the 2008 financial crisis did not radically alter the functioning of the financial systems despite the wave of criticisms of the moral hazards linked to bank bailouts (Krugman, 2009), on the other hand, economic historians point to the political economy changes promoted by the Great Depression and the two World Wars and reflected in the increasing role of the State in the economy and the construction of the Welfare State (Persson & Sharp, 2015). Clearly, the reactions to shocks and crises differ, and affect industries differently. Also, some crises are endogenous – engendered directly by the economic players as in the case of the 2008 financial crisis, while others are more (although perhaps not completely) exogenous – as in the case of natural disasters, wars, civil conflicts, and pandemics. A shock such as that wrought by the COVID-19 crisis could accentuate latent conflicts because quite suddenly imbalances and disparities across members of the same society are exacerbated, and threats which the dominant narrative has allowed to be overlooked for years are brought to light.

With all that in mind, I explore the narratives that have developed out of the “bailout conditionality” debate which emerged when countries began to discuss policy solutions to help companies hit by the COVID-19 crisis get back on track. First, I describe the narratives emerging from the bailout conditionality debates based on qualitative content analysis of reports in the international press during the crisis (from January 19 to June 7, 2020). Second, I perform a quantitative content analysis to assess the temporal dynamics of these narratives during this period.^1^

With specific reference to the bailout of large companies, three narratives stand out: the distributive justice, environmental justice, and Marxist-type anti-capitalistic narratives (Table 1).

**Table 1:** The three narratives in the Covid-19 bailout conditionality debate

Narrative	Examples
(a) Distributive justice narrative	The $2 trillion federal assistance package passed in March included hundreds of billions of dollars to prop up large corporations without questioning their commitment to workers or business practices. I understand the desire to keep businesses from failing, but doing so makes sense only if government funds are being used to support workers – *not to enrich executives and shareholders. But that’s what is happening*. Washington Post, May 14, 2020
	Now, the median FTSE 100 executive salary is £850,000, and the median bonus is £1.4m on top. *A 30 per cent cut to annual salary and the cancellation of bonus payments, however, would amount to a loss of £1.65m. Even then, our CEO would still be a member of the 0.00001 per cent. They wouldn’t go hungry and could still order whatever Lexus model they wanted (the company usually pays anyway).* The Independent, April 27, 2020
	Some businesses receiving government cash and favourable loans are continuing to *handsomely reward top executives and shareholders. Some are owned by wealthy individuals who for years have paid minimal tax personally and through their companies*. The Times, May 20, 2020
	It will have to be scrutinized further before being forwarded to the cabinet for approval. There have been heated discussions in the online and offline worlds along with insights offered by politicians, bankers, and academics on possible solutions for THAI and, more importantly, the fact that *taxpayer money worth more than 100 billion of baht could be used to fund a state-owned enterprise saddled with a long history of corruption and nepotism*. The Bangkok Post, May 18, 2020
(b) Environmental justice narrative	The economic challenge that represents the Coronavirus outbreak has also to be seen as an opportunity to undertake in the context of the *EU Green deal an urgent reorientation of the EU economy as the current crisis reveals the fragility* of a carbon-intensive system built on highly interconnected and specialised global supply chains. The Guardian, March 16, 2020
(c) Marxist-type anti-capitalist narrative	Perhaps, we need a new national flag. One that simply reads: “Profit before People”. There’s been plenty of stories about individual companies and bosses *acting despicably during the outbreak - but don’t be fooled into thinking this is about a few bad apples*. *The problem, the dysfunction, is systemic. The structures we have set in place around the economy allow the rich to sponge off the state when they need to, while simultaneously vilifying the poor and weak if they find themselves dependent on state support. It’s a satanically sick joke; hypocrisy on an epic level.* The Herald, May 7, 2020

Emphasis in Italics added.

The *distributive justice narrative* was born out of larger discussions on the rising within-country economic inequality – a debate that existed in the academic circles well ahead of the COVID-19 crisis and which was popularized in Piketty’s (2014) book *Capital in the XXI Century*. This narrative openly stigmatizes companies for non-payment of taxes and denounces their shareholders and executives for enriching themselves at a time when so many others are suffering. It also debunks all the preconceptions about the well-functioning of markets and the well-deserved payoffs of risk taking, to the point that a CEO’s salary cut is portrayed as a desirable action since the individual in question “wouldn’t go hungry” and could still afford “whatever Lexus model” she/he wanted (see Table 1(a) for examples).

The *environmental justice narrative* focuses on bailouts conditional on companies meeting certain environmental targets. Essentially, it portrays the COVID-19 crisis as an epochal opportunity to start a new green transition towards a low-carbon economy, and resonates with calls for an environmental turn in policy-making from different social groups – including the younger generations moved by the Greta Thunberg Fridays for Future initiative (see Table 1(b)).

Finally, the crisis has given space to a fairly radical *Marxist-type narrative*^2^ that questions the legitimacy of the whole capitalistic system which as the quote in Table 1(c) shows, is depicted using some extreme terminologies as a “satanically sick joke” where the “structures…in place around the economy allow the rich to sponge off the state when they need to, while simultaneously vilifying the poor and weak”.

**Figure 1:**
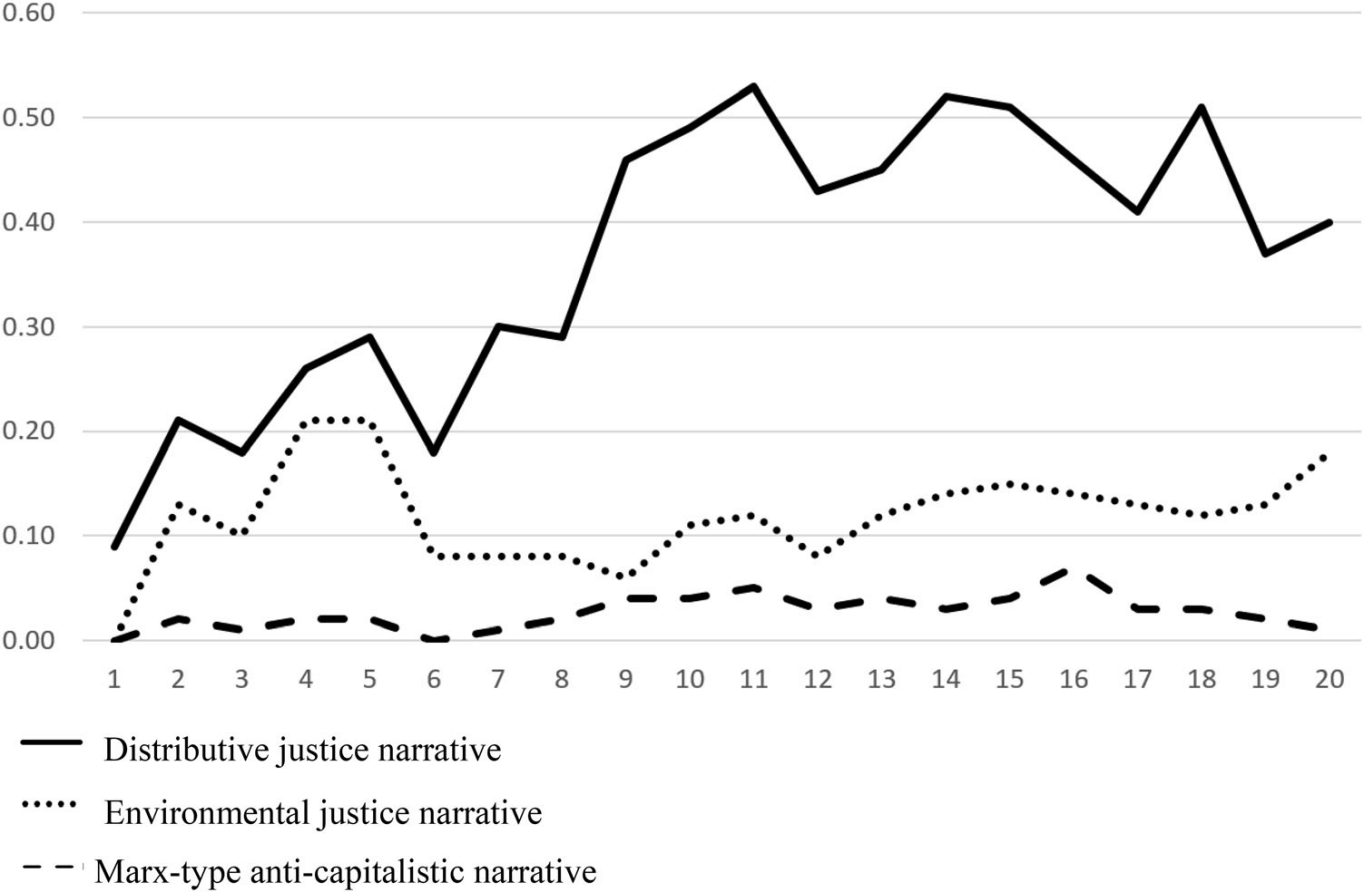
The evolution of the three narratives during the crisis. *Note*: The* Y*-axis measures the frequency of the terminologies attached to each narrative dictionary in the text, and the* X*-axis refers to the successive weeks from Jan 19 to June 7, 2020.

Next, I look at how these three narratives developed during the start-up phase of the COVID-19 crisis (20 weeks in total). Figure 1 shows that during the first 6 weeks following the start of discussion of bailout conditionalities both the distributive and environmental justice narratives increased in the press but that after that initial period the former assumed greater prominence and the relevance of the latter decreased, and for most of the remaining period of observation has remained flat apart from a slight rise in the last observed week. The Marxist-type anti-capitalistic narrative was present in the press, but was never dominant. Starting from week six, it left the stage to the distributive justice narrative. The focus on the distributive justice narrative continued through the period of observation.

## ARE WE SHIFTING IDEOLOGIES?

While the analysis of press narratives on its own is insufficient to demonstrate that we are on the brink of an epochal ideological change, it suggests that we may be becoming familiar with new truths, and that these new truths might potentially be transformative. The arguments at the core of the distributive justice narrative were present among activists (see e.g., the Tax Justice Network) and scholars (Zucman & Saez, 2019 among others) prior to the crisis but the crisis provided an opportunity for these arguments to be amplified to reach a much wider audience. Some recent research suggested that media coverage on corporate tax avoidance becomes more frequent and more negative during economic downturns, when readers display heightened sensitivity towards what they perceive to be greedy and unfair business conduct (Chen, Schuchard & Stomberg, 2019). Likewise, during the COVID-19 crisis, such distributive justice arguments have been consistently prominent in the press and have emerged in high-level policy debates, too. For instance, on April 17, 2020, the European Parliament adopted a resolution recommending that:

EU institutions and the Member States ensure that public financial support provided to firms in order to combat the economic effects of COVID-19 is conditional upon the funding being used to benefit employees and the recipient firms refraining from bonuses to the management, tax evasion, paying out dividends, or offering share buy-back schemes for as long as they receive such support. (European Parliament, 2020, 34).

In the same spirit, on April 24, 2020, EU Finance Minister Bruno Le Maire declared that “If your headquarters is located in a tax haven, it is obvious that you will not be able to benefit from public aid,” adding that “If you have benefited from the state’s cash flow, you cannot pay dividends and you cannot buy back shares” (Braun, 2020).

Other countries in Europe have announced similar conditions and even US President Donald Trump has conceded that he would “be ok with prohibiting companies that receive federal assistance during the coronavirus pandemic from using that money for stock buybacks in the future” (Reinicke, 2020).

Certainly, a discussion about the need to avoid a “lemon socialism” scenario, where taxpayers are asked to absorb the losses of failing companies, is not new to this COVID-19 crisis: it was an issue of concern during the 2008 financial crisis, too (Krugman, 2009), and back then some voices were raised about the moral inappropriateness of allowing bailed out banks and financial institutions to award dividends to shareholders or bonuses to executives. However, in concrete terms, very little was achieved on this front at that time (see Acharya, Gujral, Kulkarni, & Shin, 2011; Jabko & Massoc, 2012), but these arguments could achieve more traction in the context of the COVID-19 crisis, given the severe, pervasive, and enduring impacts on the real economy.

The relevance of these seemingly changing narratives might be signaling a small step towards an ideological shift in which the cognitive frames of the general public and the electorate may become more open to scrutiny of companies’ dividend and bonus policies (hereafter distributive policies) and more averse to acceptance by society of tax avoidance and the accumulation of excessive wealth by members of the corporate elite (e.g., majority shareholders and executives). These narratives may even potentially intensify in the future as governments will have to decide whether to continue to bailout companies in the aftermath of the crisis and which types of fiscal policies are better suited for containing inequality, which may prompt debates about the need to raise marginal tax rates for the wealthy or on capital gains (Dietsch et al., 2020). Such continuing debate may keep the general public alert and trigger demands for the accumulation of wealth to not be at the expense of other stakeholders (e.g., workers, communities) or the environment. Should there be some “normalization” of these kinds of expectations among the general public, this could leave ample room to redesign policies to address sustainability grand challenges other than pandemics. For instance, it might increase consensus about the need to explore a new policy space where public subsidies to companies and market-based incentives such as competitive bidding schemes must be anchored firmly to companies’ human rights and environmental track records. It might also promote related policies banning or disincentivizing companies from redistributing their gains unless they show improved social and environmental performance or limit systematic state support to companies with aggressive tax planning. Perhaps the bailout conditionality debate provides another opportunity to set the ground for more courageous policy-making in one or more of these directions which is what is needed most to ensure that a greater difference is marked between our future and Daniel Defoe’s times.

Finally, ideological shifts of the kind discussed above could modify the expectations of MNEs stakeholders – especially workers and external audiences including consumers, the press, and the general public. In that scenario, MNEs possibly might in the future have to face greater scrutiny of their distributive policies, and demands for solid justifications for payments of dividends to shareholders or bonuses to executives despite the workers involved in their value chains for instance not being guaranteed minimum wages. It is well known that MNEs have already adopted sophisticated impression management strategies to deal with multiple accountability requests on a complex range of social and environmental issues (Fabrizio & Kim, 2019) and that CEOs are becoming activists themselves by adopting an open stance towards some of the apparent aberrations of contemporary Western societies – as in the case of racial or LGBQT+ discrimination (Chatterji & Toffel, 2019). Nonetheless, it is less clear how such advancements might contribute to resolving the tensions at the core of capitalism which cannot be addressed simply by the addition of a new policy to augment the lists of MNE “good deeds”. Sociologist Johan Bellamy Foster reframes Marx’s idea of capitalism as a production system that “is thoroughly wasteful with human material….so that it loses for society what it gains for the individual capitalist” (Marx, Vol. III pp. 90–92 quoted in Foster, 2020, p. 183). Hence, if we were to ask MNEs to play their part in reforming capitalism such that wealth accumulation at the top of the value chain (shareholders, executives) should not be at the expense of those at the bottom (workers, children, vulnerable groups, etc.), we would be asking for a radical reform of corporate governance that would undoubtedly need to exceed what social responsibility policies currently do. Irrespective of whether the COVID-19 crisis will lead to such a radical transition at this time, it seems to me that current and future global corporate leaders should focus seriously on familiarization with distributive justice and other similar narratives in order to be prepared to act responsibly when the next crisis strikes.

## Notes

1. Quantitative content analysis has been used to document trends in rhetorical strategies over time. I used ad hoc dictionaries (available upon request) and LIWC software to analyze the press. The selection of press was made using the Nexis Uni database to identify “Newspaper” articles based on keyword search “Coronavirus OR Covid AND bailout”. The search included over 12,000 articles (amounting to 21,105,655 words) written in English. While this selection includes newspapers from non-English speaking countries if they have newspapers published in English, it excludes other languages which may bias the universe towards more “Western” views on the matter. Also, the analysis does not consider outlets other than newspapers (e.g., blogs, industry reports). Hence, the results presented in this note need to be considered in light of these caveats.
2. It has to be acknowledged here that this narrative reflects how Marx’s core ideas were popularized, not his own thinking. As Krätke (2020, p. 20) puts it, Marx “did not criticize capitalism as a system of injustice or condemn it as the source of all evils; he saw capitalism not as a wrong track leading mankind astray from its ‘true’ destination, but as a necessary and largely progressive stage in human history” and he believed rather that “capitalism would come to an end because of its inherent tendencies to self-destruction”.
